# A combination weighting method for debris flow risk assessment based on t-distribution and linear programming optimization algorithm

**DOI:** 10.1371/journal.pone.0303698

**Published:** 2024-06-14

**Authors:** Li Li, Hanjie Lin, Yue Qiang, Yi Zhang, Shengchao Hu, Hongjian Li, Siyu Liang, Xinlong Xu

**Affiliations:** Civil Engineering College, Chongqing Three Gorges University, Wanzhou, Chongqing, China; ICIMOD: International Centre for Integrated Mountain Development, NEPAL

## Abstract

Debris flow risk assessment can provide some reference for debris flow prevention and control projects. In risk assessment, researchers often only focus on the impact of objective or subjective indicators. For this purpose, this paper proposed a weight calculation method based on t-distribution and linear programming optimization algorithm (LPOA). Taking 72 mudslides in Beichuan County as an example, this paper used analytic hierarchy process (AHP), entropy weight method (EWM) and variation coefficient method (VCM) to obtain the initial weights. Based on the initial weights, weight intervals with different confidence levels were obtained by t-distribution. Subsequently, the final weights were obtained by LOPA in the 90% confidence interval. Finally, the final weights were used to calculate the risk score for each debris flow, thus delineating the level of risk for each debris flow. The results showed that this paper’s method can avoid overemphasizing the importance of a particular indicator compared to EWM and VCM. In contrast, EWM and VCM ignored the effect of debris flow frequency on debris flow risk. The assessment results showed that the 72 debris flows in Beichuan County were mainly dominated by moderate and light risks. Of these, there were 8 high risk debris flows, 24 medium risk debris flows, and 40 light risk debris flows. The excellent triggering conditions provide favorable conditions for the formation of high-risk debris flows. Slightly and moderate risk debris flows are mainly located on both sides of highways and rivers, still posing a minor threat to Beichuan County. The proposed fusion weighting method effectively avoids the limitations of single weight calculating method. Through comparison and data analysis, the rationality of the proposed method is verified, which can provide some reference for combination weighting method and debris flow risk assessment.

## 1 Introduction

The debris flow is a terrible natural disaster occurring mainly in mountainous areas [1]. Nearly 66.7% of China’s land area is mountainous, making it highly vulnerable to debris flow disasters in the global context [2]. Each year, debris flow disasters cause hundreds of deaths and nearly 1,000 injuries, resulting in economic losses totaling up to 2 billion dollars [2]. Therefore, performing a risk assessment for high-risk debris flow disaster areas can take measures in advance to reduce their destructive capacity.

Currently, debris flow risk assessment is mainly divided into multi-factor superposition method and numerical simulation method. The multi-factor superposition method is mostly used to solve multi-criteria decision-making problems. This method is mainly used to determine the potential danger level of debris flow occurring. In contrast, numerical simulation methods are mostly used to solve the movement characterization of debris flows occurring in gullies and valleys. The widely used multi-factor superposition methods mainly include entropy weight method (EWM) [3], gray correlation method [4], etc. And the widely used numerical simulation methods mainly include FLO-2D [5], Mass-Flow [6], etc. Combining the multifactor superposition method with numerical simulation methods can obtain more comprehensive assessment results [7]. In addition, with the rapid development of risk assessment methods based on machine learning, multifactor superposition is often used as a key parameter for tuning the algorithm [8]. Therefore, the multifactor superposition method remains one of the key issues to be explored in debris flow risk assessment.

The study showed that how to determine the weights in debris flow risk assessment needs to be further investigated [9]. Tan et al. [10] used analytic hierarchy process (AHP) to determine the metric weights of Wudongde Dam debris flow risk assessment. Gu et al. [3] used EWM to determine the metric weights of debris flow risk assessment. Ren et al. [11] utilized the variation coefficient method (VCM) to determine the weights of assessment factors. Wang et al. [12] used AHP to construct a risk assessment system of influence factors. Cai et al. [13] combined AHP and Radial basis function (RBF) neural networks to recalculate the weights of metrics. For multicriteria decision-making problem in the above study, the calculation methods of weights mainly included subjective or objective decision-making methods. The subjective decision-making method focuses on determining the weights by experts scoring the factors. In contrast, the objective decision-making method depends on the relationship between the data to calculate the factor weights. However, due to the different focuses of the two methods, the results obtained by the methods often have large deviations. Therefore, how to apply the weight calculation methods more rationally has always been a key concern [14].

In recent years, with some combination weighting methods were proposed to provide new directions for multicriteria decision-making problems. Liu et al. [15] combined the metric weights of AHP and EWM to obtain new weight values. The results showed that the improved method provided more reasonable results compared to HAP and EWM. Chen [16] combined the weight values of EWM, AHP, and technique for order preference by similarity to an ideal solution (TOPSIS) method to obtain new metric weights. The results showed that the proposed method can select the appropriate construction material suppliers more efficiently. Akram et al. [17] combined stepwise weighted assessment ratio analysis (SWARA) with complex offset proportion rating assessments (COPRAS) to propose a generalized MAGDM framework. The results showed that the improved method is feasible, effective and robust after comparative analysis. The above analyses all showed that the combined weighing values combining subjective and objective influences provided reasonable evaluation results.

However, the current combination weighting methods applied to debris flows still have some limitations. Li et al. [18] indicated that researchers often focus only on the impact of objective or subjective indicators, and that the impact of both needs to be considered together. For simultaneously considering the researcher’s intuitive recognition in the field survey and respecting the laws of objective data, it is necessary to combine subjective and objective decision-making methods [18]. Li et al. [19] proposed a debris flow risk assessment method based on combination weights of probability analysis (CWPA) in 2022. This method combined the weights of the equal weight method, VCM and EWM to construct the weight intervals. Subsequently, the final weights were calculated by probabilistic methods. The results showed that CWPA is feasible by comparing it with the actual results. However, in the process of calculating weights, the equal weight method results show that all factors have the same weights. The fact is that for debris flows in different areas, the importance of assessment metrics is different. In addition, CWPA only took the maximum and minimum values as upper and lower limits of weight intervals. In contrast, the interval generated by t-distribution is suitable for small samples [20] and more resistant to interference [21]. Therefore, the t-distribution is more advantageous compared to the CWPA for the single-trench evaluation of debris flows. On the other hand, the weights calculated by the CWPA are consistent across the study area. For this reason, this paper introduced a linear programming optimization algorithm (LPOA) to dynamically obtain values within weight intervals. The LPOA can obtain more refined results. Sung et al. [22] proposed a scheme for deploying large satellites based on a dynamic model of LPOA. The results showed that the model is effective in saving program costs. Hashemi-Amiri et al. [23] constructed a bi-objective multi-level perishable food supply chain network based on LPOA. The results showed that this method can optimize the network economic effects and improve the raw material procurement reliability. Pilotti et al. [24] proposed a model for the design and operation of hybrid photovoltaic power plants based on a LPOA. The results showed that the improved model can achieve similar or better levels of dispatchability at lower power costs. The above studies have proved that the current LPOA has been widely used in various fields and it is a feasible method.

Therefore, this paper proposed a model for debris flow risk evaluation applicable to small samples based on t-distribution and LPOA. Initial weights were calculated by using AHP, EWM and VCM. The AHP is a simple, feasible and subjective decision-making method [25]. In contrast, the decision-making process of EWMs and VCM relies exclusively on the objective data itself. Fusion of the 3 methods can provide a comprehensive assessment result. Calculate the mean and variance of the weights obtained by the different methods, and subsequently calculate the weight intervals according to t-distribution mathematical principles. Use the above intervals as constraints, and set the maximum risk score as objective function. Based on the objective function and constraints, the final weights were obtained by using the single objective criterion of LOPA.

This paper aims to (1) Propose a combination weighting method based on t-distribution and LOPA. (2) The proposed method was applied to the debris flow risk assessment, and comparing to verify the effectiveness of this paper’s method. In Section 2, this paper introduced the study area and the dataset. In Section 3, this paper described the main methods used and the proposed method. In Section 4, this paper presented results of this study including sort of risk scores and the degree of debris flow risk in Beichuan County. In Section 5, the paper verified the validity of the final results by analyzing the data and comparing the results, and discussed the limitations of this paper and future research directions. In section 6, the paper concluded this study.

## 2. Study area and dataset

### 2.1 Study area

The paper used 72 debris flow gullies in Beichuan County as the study object. Due to the "5.12" Wenchuan Earthquake, a large number of loose deposits were generated in this area. Also, due to the large difference in topographic elevation, surface runoff was concentrated, and gully erosion was serious. In addition, due to the location on the Eurasian seismic belt, the frequency of large and small earthquakes increases the possibility of debris flow disasters in the area.

#### 2.1.1 Topography and geomorphology

Beichuan County is located in Sichuan Province of western China. The whole topography can be divided into high, medium and low mountainous areas. The high mountainous areas occupy 46.5% of the total area in Beichuan County. Due to the high elevation and harsh climate of the alpine region, the area is sparsely populated and mostly covered with virgin forests. Low mountainous areas occupy 16.0% of the total area of Beichuan County. Due to the close proximity of the low mountains to the Sichuan Basin, the topography is relatively gentle and geologic risks are developed less frequently.

#### 2.1.2 Hydrographic condition

The Jian River is the main river in Beichuan County, and is a first-class tributary of the Fu River. This river has a total length of 47.9km, a watershed area of 455.80km^2^, a natural drop of 203m, and an average specific drop of 4.2‰. In addition, this river has a multi-year average runoff of 102.7m^3^/s, an annual average runoff total of 3.257 billion m^3^, and an average annual sand transport of 4–5 million tons.

#### 2.1.3 Stratigraphic lithology

The Paleozoic Cambrian, Silurian, Devonian, Carboniferous strata and the Cenozoic Quaternary loose stacked strata are distributed in the study area. The stratigraphic lithology mainly includes the upper Sinian system (Z_2_), Lower Cambrian System (*ε*_1_), middle Siluran System (S_2_), upper Siluran system (S_3_), Devonian system (D), Carboniferous system (C), Permian system (P), Lower Triassic system (T_1_), Quaternary middle Pleistocene system (Q_2_), Quaternary upper Pleistocene system (Q_3_) and Quaternary Holocene system (Q_4_).

### 2.2 Dataset

Huo [26] counted 42 papers in related fields to summarize the most used assessment metrics. Therefore, this paper uses the more frequent 6 assessment metrics to make the results comparable.

The debris flow scale (*X*_*1*_). The larger *X*_*1*_ represents the larger volume of produced loose material, and the more destructive in the event of a debris flow disaster [14].

The basin area (*X*_*2*_). The *X*_*2*_ reflects the basin’s catchment and sand production [14]. It relates to the hydropower conditions and physical source conditions of the debris flow.

The basin cut density (*X*_*3*_). The *X*_*3*_ represents the ratio of total gully length to basin area in the region [14]. It indirectly reflects the regional sand production and the degree of rock weathering.

The basin relative elevation difference (*X*_*4*_). The *X*_*4*_ denotes the dynamical conditions of the debris flow. It can reflect the energy of the debris flow.

The main gully length (*X*_*5*_). The *X*_*5*_ determines the recharge length and the ability to accept solid loose material. And in terms of frequency of use, this indicator is much greater than the length of the sediment recharge segment.

The debris flow frequency (*X*_*6*_). The *X*_*6*_ is the number of debris flows occurring per 100 years. It is one of the factors that most directly affects debris flow risk.

**Fig 1:**
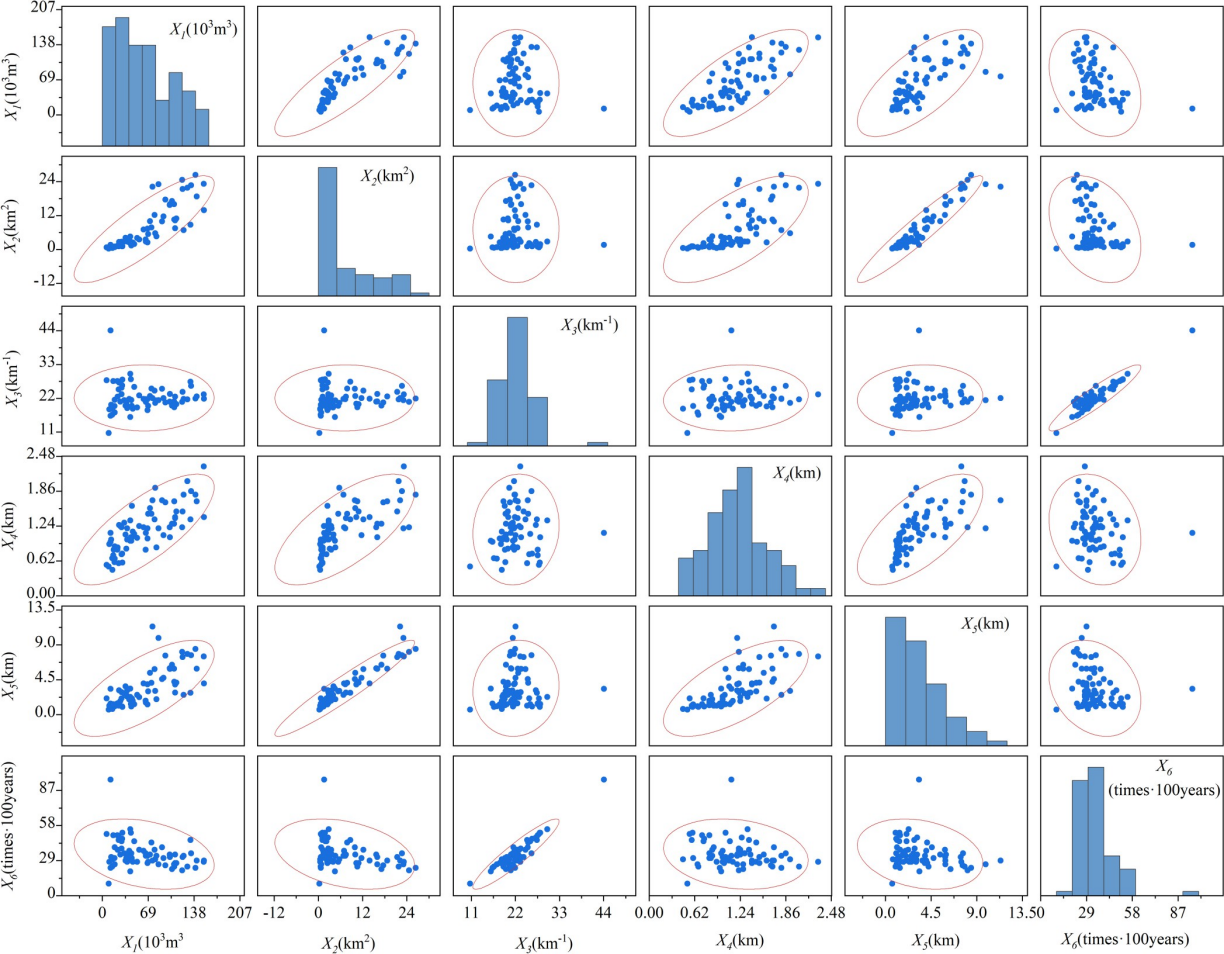
The distribution and correlation of 72 debris flows data.

Table 1 shows the basic survey data of 72 mudslides in Beichuan County [14]. Fig 1 shows the interrelationships and distribution of the data from Table 1.

**Table 1:** The basic data of 72 debris flows in Beichuan County.

Samples	X_1_(10^3^m^3^)	X_2_(km^2^)	X_3_(km^-1^)	X_4_(km)	X_5_(km)	X_6_
#1	44.65	2.5	19.32	1.6	2.06	29
#2	152.83	13.9	21.85	1.4	4.03	29
#3	109.66	10.3	20.21	1.4	3.89	27
#4	73.9	7.8	25.17	1.46	4.1	44
#5	42.19	4.5	15.89	0.98	3.35	20
#6	78.51	12.2	24.5	1.36	5.88	38
#7	42.97	1.8	28.17	1.04	1.38	52
#8	71.87	10	22.71	1.56	5.39	34
#9	48.06	1.9	25.68	1.1	1.36	45
#10	45.95	2.4	18.63	1.14	1.78	28
#11	12.29	1.6	44.06	1.12	3.32	96
#12	15.31	0.5	18.6	0.46	0.73	30
#13	18.53	0.8	17.38	0.66	1.09	26
#14	109.37	7.5	22.55	1.38	2.86	34
#15	81.51	4.6	21.33	0.86	1.99	33
#16	127.85	21.8	21.67	2.04	7.82	24
#17	79.63	5.7	20.47	1.92	3.05	30
#18	122.46	6.8	21.79	1.8	2.55	33
#19	152.47	23.2	23.24	2.3	7.49	28
#20	108.22	10.6	19.05	1.68	4.25	24
#21	101.65	16	23.43	1.04	5.89	32
#22	55.62	3.5	22.94	1.24	2.25	38
#23	12.39	0.7	16.29	0.96	1.43	23
#24	13.48	0.7	19.86	1	1.36	32
#25	142.01	18.7	22.6	1.68	5.91	29
#26	16.56	0.9	27.56	0.72	1.36	50
#27	22.94	1.2	22.5	0.86	1.45	38
#28	18.33	1	19.5	0.6	1.32	31
#29	18.77	0.8	21.5	0.88	1.15	40
#30	101.41	15.7	20.89	1.22	5.97	34
#31	120.62	21.4	24.07	1.5	7.42	32
#32	132.74	8.7	27.37	1.36	2.83	46
#33	89.77	9.9	25.09	1.7	4.59	40
#34	22.55	1.2	27.58	0.82	1.45	50
#35	29.85	1.1	26.18	0.75	1.05	47
#36	94.94	17.6	20.65	1.66	7.62	25
#37	29	2.7	23.33	1.22	2.89	39
#38	25.23	2.6	27.12	1.26	3.13	48
#39	17.07	0.8	19.38	0.82	1.22	31
#40	14.73	0.6	16.67	0.67	0.99	24
#41	28.98	2.2	25.45	0.74	2.09	45
#42	9.65	0.3	10.67	0.52	0.66	10
#43	6.3	0.7	27.86	0.55	2.09	51
#44	37.46	2.8	18.79	1	2.35	28
#45	30.05	1.1	28.09	0.59	0.99	52
#46	10.66	0.5	18.4	0.92	1.15	28
#47	43.62	1.4	21.43	0.98	1.06	35
#48	24.71	1.1	24.91	0.58	1.15	46
#49	44.38	1.8	19.56	1.04	1.35	31
#50	36.11	3.1	20.1	0.84	2.54	31
#51	65.25	3.5	21.74	0.82	1.8	35
#52	120.07	24.6	20.84	1.22	8.1	21
#53	65.45	2.8	20.82	1.12	1.56	33
#54	35	4.1	19.63	1.09	3.64	29
#55	75.47	22.2	22.05	1.7	11.36	29
#56	37.32	3.6	21.25	1.2	3.12	34
#57	61.93	7	21.8	1.22	4.02	37
#58	40.61	2.5	21.8	1	1.97	37
#59	84.65	23.1	21.34	1.2	9.88	26
#60	52.41	4	18.98	1.03	2.56	28
#61	108.08	16	20.99	1.28	5.91	26
#62	91.94	11.7	22.03	1.08	4.74	31
#63	68.91	5.4	19.57	1.46	3.02	28
#64	133.63	22.7	26.04	1.86	7.66	35
#65	68.08	2.5	18.12	1.02	1.32	27
#66	26.93	1.5	20.93	1.24	1.75	34
#67	33	2.8	21.36	1.3	2.75	35
#68	67.82	7.3	21.68	1.2	3.91	33
#69	140.26	26.4	21.93	1.8	8.46	23
#70	42.18	2.8	29.96	1.34	2.29	55
#71	111.43	10.9	20.11	1.5	4.12	27
#72	104.81	17.1	19.64	1.28	6.43	22

Due to the different international units of each assessment factor, which can affect the results, it is necessary to normalize the decision matrix. Therefore, this paper uses the data in Table 1 as the decision variable matrix *Z*, where $Z=\left\{z_{ij}\right\}(i=1,2,\ldots ,\text{m};j=1,2,\ldots ,\text{n})$. m is the number of debris flows, in this paper m = 72; n is the number of assessment metrics, in this paper n = 6. This paper uses min-max normalization to handle the evaluation metrics, which were mapped into interval [0, 1], as shown in formula (1).

$${\bar{z}}_{ij}=\frac{z_{ij}-\min\left(z_{j}\right)}{max\left(z_{j}\right)-min\left(z_{j}\right)}$$ (1)

where *z*_*ij*_ is the value of the *jth* evaluation indicator for the *ith* debris flow; ${\bar{Z}}_{ij}$ is the normalized value of *z*_*ij*_; *max* (*z*_*j*_) is the maximum value of the *jth* indicator; min (*z*_*j*_) is the minimum value of the *jth* indicator.

## 3. Methods

### 3.1 Initial weight calculation methods

#### 3.1.1 Analytic Hierarchy Process (AHP)

The AHP is a subjective decision-making tool that decomposes complex problems into simple criteria. The AHP includes 3 principles: problem decomposition, comparative judgment, and relative importance (ranked synthesis). The steps for using AHP in this paper are as follows:

(1) The AHP was applied to construct the debris flow risk assessment index system (Table 2). This system mainly includes a top layer (A), a middle layer (B), and an indicator layer (C). This paper determined the final debris flow risk assessment system by referring to related literature [27], as shown in Table 1.

**Table 2:** AHP assessment system.

Top layer (A)	Debris flow risk assessment system	
Middle layer (B)	Historical activity	Potential formation conditions
Indicator layer (C)	Debris flow scale (*X*_*1*_) Debris flow frequency (*X*_*6*_)	Basin area (*X*_*2*_) Basin cut density (*X*_*3*_) Basin relative elevation difference (*X*_*4*_) Main gully length (*X*_*5*_)

(2) Establish a corresponding assessment indicator system. If the factors B_1_, B_2_ …, B_n_ have a relationship with the factor A_k_ in the previous layer A, it can be represented by a judgment matrix (Table 3). Because this paper has the middle and indicator layers, many judgments need to be performed. Instead, the results of intercomparison between different factors in the same layer were determined according to Table 4.

**Table 3:** Comparison matrix.

A_k_	B_1_	B_2_	B_3_	…	B_n_
B_1_	b_11_	b_12_	b_13_	…	b_1n_
B_2_	b_21_	b_22_	b_23_	…	b_2n_
…	…	…	…	…	…
B_n_	b_n1_	b_n2_	b_n3_	…	b_nn_

In the above table, if A is the top layer, B is the middle layer.

**Table 4:** Scale of AHP.

Degree of preference	Definitions	Explanation
1	Equally Important	Both criteria are equally important or both the factors have same effect on occurrence of debris flow
3	Moderately Important	One factor is more effective as compared to the other factor
5	Highly Important	One factor affects highly as compared to the other factor
7	Very Highly Important	A factor is highly dominated over other
9	Extremely Important	A factor has highest possibility of affecting the occurrence of landslide over other factor
2,4,6,8	Intermediate Values	If a compromise between two factors is required, intermediate values can be used

This paper scores the metrics through the assessment criteria shown in Table 4, with reference to the relevant literature [27] and the field survey results. The judgment matrices *A*−*B* for top layer to middle layer, and *B*_1_−*C* and *B*_2_−*C* for middle layer to indicator layer are obtained respectively.


$$A-B=\left[\begin{array}{cc} 1 & 1/2\\ 2 & 1 \end{array}\right],B_{1}-C=\left[\begin{array}{c} 1\;1\\ 1\;1 \end{array}\right]$$

$$B_{2}-C=\left[\begin{array}{cccc} 1 & 1 & 2 & 2\\ 1 & 1 & 1 & 1\\ 1/2 & 1 & 1 & 1\\ 1/2 & 1 & 1 & 1 \end{array}\right]$$

(3) Calculate the largest characteristic root *λ*_*max*_ and the corresponding feature vectors of the judgment matrices. The feature vectors were normalized to obtain the weights *W*, which are the sorting weights of each metric in the same layer to the metrics in the previous layer. To ensure the reasonableness of AHP results, the consistency test of the matrix is required when the matrix order exceeds 2. The consistency ratio (*CR*) for calculating the judgment matrix is shown in formula (2).


$$CR=\frac{\left(\lambda_{max}-n\right)/(n-1)}{RI}$$ (2)

Where *n* is the number of matrix orders, the *RI* value can be obtained by searching the table. When *CR* < 0.1, the consistency of the matrix is considered acceptable; otherwise, the values of judgment matrix need to be adjusted.

The corresponding results for the *A*–*B*,*B*_1_ − *C*,*B*_2_ − *C* matrices are shown in Table 5.

**Table 5:** The corresponding result for matrices.

matrix	*W*	*λ_max_*	RI	CR	Consistency testing
*A* − *B*	*W* = [0.414,0.586]^*T*^	2.061	—	—	—
*B*_1_ − *C*	*W* = [0.500,0.500]^*T*^	2.000	—	—	—
*B*_2_ − *C*	*W* = [0.345,0.244,0.205,0.205]^*T*^	4.061	0.882	0.023	Pass

The results in Table 5 were collated to obtain the final AHP weight, as shown in Table 6.

**Table 6:** The final AHP weights for each metric.

Assessment metrics	Historical activity	Potential formation conditions	AHP weight
Weight of middle layer	0.414	0.586	—
X_1_	0.5	0	0.207
X_2_	0	0.345	0.203
X_3_	0	0.244	0.143
X_4_	0	0.205	0.120
X_5_	0	0.205	0.120
X_6_	0.5	0	0.207

#### 3.1.2 Entropy Weight Method (EWM)

The principle of EWM is to determine the dispersion degree of evaluation factors by the magnitude of information entropy. EWM is an unstructured, objective decision-making method which can reduce subjectivity in evaluation methods. The specific steps for using EWM in this paper are as follows:

(1) The information entropy value *H*_*j*_ of each assessment metric was calculated based on the normalized data, as shown in formula (3).


$$H_{j}=-\frac{1}{\text{lnm}}\sum_{i=1}^{m}\frac{\tilde{z_{\text{ij}}}}{\sum_{i=1}^{m}{\tilde{z}}_{\text{ij}}}\text{ln}\frac{\tilde{z_{\text{ij}}}}{\sum_{i=1}^{m}\tilde{z_{\text{ij}}}}$$ (3)

Where *H*_*j*_ is the information entropy value of the *jth* metric. The smaller *H*_*j*_ of assessment metrics represents the greater role played in the assessment.

(2) The entropy weight $w_{j}^{EWM}$ of each assessment metric was calculated, as shown in formula (4).


$$w_{j}^{EWM}=\frac{1-H_{j}}{n-\sum_{j=1}^{n}H_{j}}$$ (4)

Where $w_{j}^{EWM}$ is the entropy weight of the *jth* metric; n is the number of assessment metrics, in this paper n = 6.

The results of the above calculations are shown in Table 7.

**Table 7:** The final EWM weights for each metric.

Assessment metrics	*H*_*j*_	$w_{j}^{EWM}$
X_1_	0.935	0.187
X_2_	0.873	0.366
X_3_	0.988	0.035
X_4_	0.964	0.104
X_5_	0.916	0.262
X_6_	0.977	0.066

#### 3.1.3 Variation coefficient method (VCM)

The VCM is an objective weighting method based on statistical methods for calculating the change degree of metrics [28]. It can objectively reflect the change information of factors. A larger variance gap represents a larger gap between the actual value of the indicator and the desired target value. Therefore, the weight of the metrics is greater when the variance gap is greater. The specific steps for using VCM in this article are as follows:

(1) The mean ${\bar{z}}_{j}$ and the standard deviation *S*_*j*_ of assessment metrics will affect the magnitude of variation coefficients simultaneously. Therefore, it is needed to calculate ${\bar{z}}_{j}$ and *S*_*j*_ firstly, as shown in formula (5).


$$\left\{\begin{array}{r} {\bar{z}}_{j}=\frac{1}{n}\overset{n}{\underset{i=1}{\sum }}z_{ij}\\ S_{j}=\sqrt{\frac{\sum_{i=1}^{n}{\left(z_{ij}-{\bar{z}}_{j}\right)}^{2}}{n-1}} \end{array}\right.$$ (5)

Where, ${\bar{z}}_{j}$ is the mean of the *jth* indicator; *S*_*j*_ is the standard deviation of the *jth* indicator; n is the number of assessment metrics, in this paper n = 6.

(2) The variation coefficient *v*_*j*_ of the assessment metrics is the ratio of the *S*_*j*_ to the ${\bar{z}}_{j}$, as shown in formula (6).


$$v_{j}=\frac{S_{j}}{\bar{x_{j}}}$$ (6)

Where, *v*_*j*_ is the variation coefficient of the *jth* indicator.

(3) Normalizing the Normalizing the variation coefficients is to obtain the weights *w*_*j*_ of the VCM, as shown in formula (7).


$$w_{j}^{VCM}=\frac{v_{j}}{\sum_{j=1}^{n}v_{j}}$$ (7)

Where, $w_{j}^{VCM}$ is the EWM weight of the *jth* indicator.

The results of the above calculations are shown in Table 8.

**Table 8:** The final VCM weights for each metric.

Assessment metrics	${\bar{z}}_{j}$	*S*_*j*_	*v*_*j*_	$w_{j}^{VCM}$
X_1_	0.418	0.276	0.659	0.204
X_2_	0.143	0.15	1.045	0.323
X_3_	0.732	0.116	0.158	0.490
X_4_	0.587	0.189	0.321	0.099
X_5_	0.227	0.163	0.717	0.221
X_6_	0.229	0.077	0.334	0.103

### 3.2 Proposed method

This paper determined the final weights based on the t-distribution and LPOA. From a probabilistic point of view, the t-distribution has the property of being applicable to small samples [20]. However, this method can only generate weight intervals. The LPOA, as an important component of operations research, is nowadays widely used in optimization problems of aerospace [22], food supply chains [23] and energy supply [24]. The basic principle is setting objective function and constraints, so as to solve for the extreme value of the objective function within the constraints. Depending on the number of objective functions, linear programming problems can be divided into multi-objective and single-objective linear programming. Considering the debris flow risk only solving for a very large risk value, this paper adopted the single-objective linear programming optimization algorithm. The specific steps of the proposed method are as follows:

(1) Calculate the mean weights of AHP, EWM, and VCM in the *jth* metric, as shown in formula (8).


$$\bar{w_{j}}=\frac{w_{j}^{1}+w_{j}^{2}+\cdots +w_{j}^{l}}{n}$$ (8)

Where, $\bar{w_{j}}$ is the mean weight of the jth metric; $w_{j}^{l}$ is the weight of the *lth* weight calculation method in the *jth* metric. *n* is the number of weight calculation methods, and in this paper *n* = 3.

(2) Calculate the variance of AHP, EWM and VCM in the *jth* metric, as shown in formula (9).


$$S_{j}^{2}=\frac{1}{n-1}\left[{\left(w_{j}^{1}-\bar{w_{j}}\right)}^{2}+{\left(w_{j}^{2}-\bar{w_{j}}\right)}^{2}\cdots +{\left(w_{j}^{l}-\bar{w_{j}}\right)}^{2}\right]$$ (9)

Where, $S_{j}^{2}$ is the variance of weights for the *jth* item indicator.

(3) According to the principle of t-distribution, the weight interval of the *jth* metric was calculated, as shown in formula (10).


$$\left(\bar{w_{j}}-t_{\alpha /2}\frac{S_{j}}{\sqrt{n}},\bar{w_{j}}+t_{\alpha /2}\frac{S_{j}}{\sqrt{n}}\right)$$ (10)

Where, *S*_*j*_ is the standard deviation of the *jth* metric, $S_{j}={\left(S_{j}^{2}\right)}^{0.5}$. When the confidence levels are 0%, 60%, 70%, 80%, 90%, and 95%, the *t*_*α*/2_ is 0.711, 0.896, .119, 1.415, 1.895, and 2.365, respectively.

(4) Set the maximum value of debris flow risk score (*P*_*i*_) as the objective function, as shown in Eq (11).


$$MaxP_{i}=\sum_{j=1}^{n}w_{ij}^{final}{\bar{z}}_{ij}$$ (11)

Where, *P*_*i*_ is the risk score of the *ith* debris flow; $w_{ij}^{final}$ is the final weight of the *jth* indicators for the *ith* debris flow.

(5) The constraints of this paper were set according to formula (12), as shown in formula (10).


$$restrictive\;condition=\left\{\begin{array}{c} \bar{w_{j}}-t_{\alpha /2}\frac{S_{j}}{\sqrt{n}}\ll w_{ij}^{final}\ll \bar{w_{j}}+t_{\alpha /2}\frac{S_{j}}{\sqrt{n}}\\ \sum_{1}^{j}w_{ij}^{final}=1 \end{array}\right.$$ (12)

(6) Constantly iterating under the constraints to find the feasible solution weights for *jth* indicators of the *ith* debris flow respectively.

### 3.3 Risk assessment

The final weights were brought into formula (13) to obtain the debris flow risk score (*P*_*i*_).


$$P_{i}=\sum_{j=1}^{n}w_{ij}^{final}{\bar{z}}_{ij}$$ (13)

This paper referred to relevant literature to give a risk classification table corresponding to Eq (9) (Table 9).

**Table 9:** The debris flow risk classification.

Risk level	Level description	Range of *P*_*i*_
A	slight risk	Pi≤0.35
B	Medium risk	0.35<Pi≤0.6
C	High risk	0.6<Pi≤0.85
D	extreme risk	Pi>0.85

**Fig 2:**
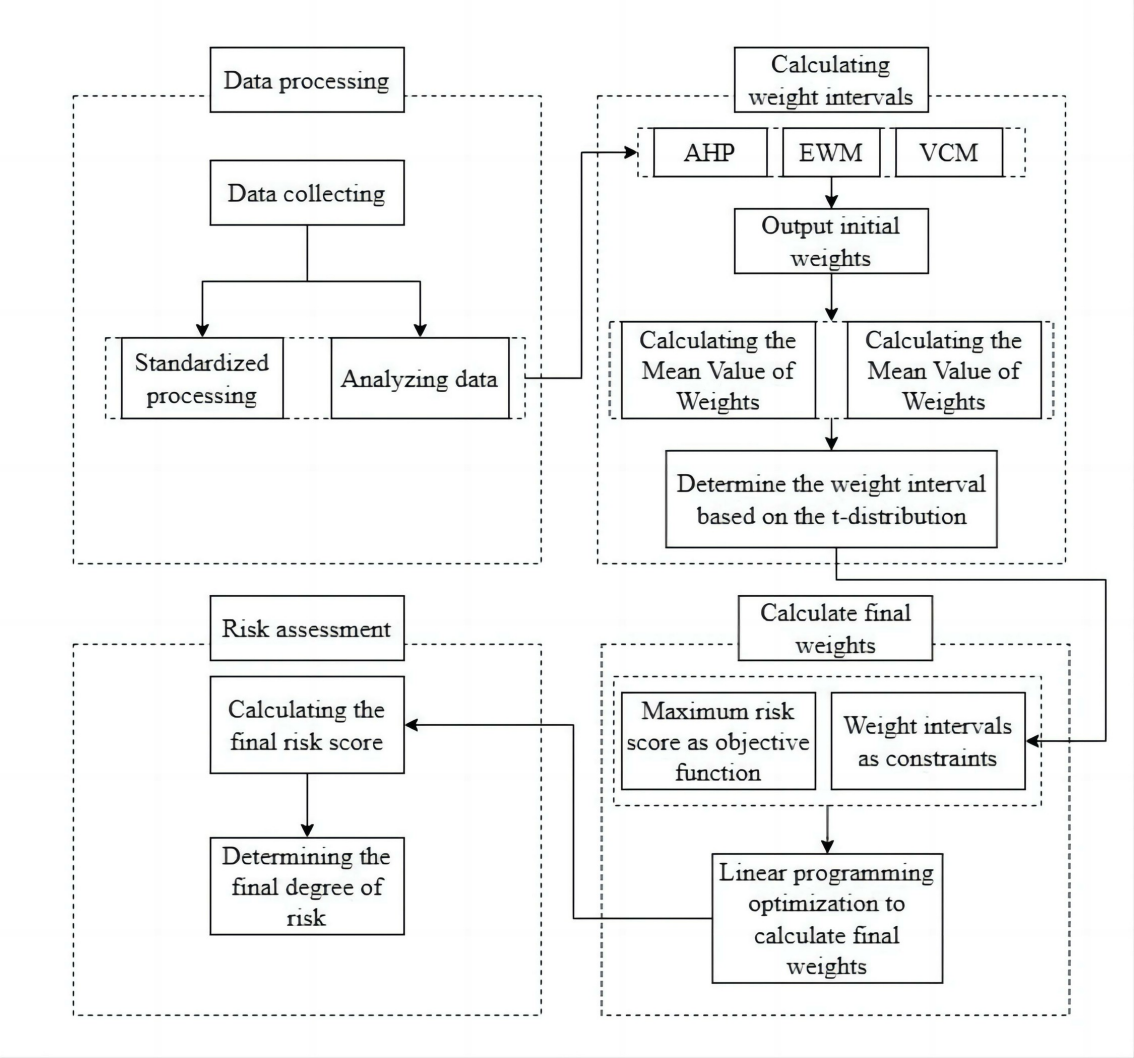
A combination weighting method for debris flow risk assessment based on t-distribution and linear programming optimization algorithm.

Firstly, this paper normalized the data of 72 debris flows. Initial weights were calculated by the AHP, EWM and VCM. Subsequently, calculating the mean and variance of initial weights, the initial weights were fused through the t-distribution principle. Setting the objective function and constraints for linear programming optimization algorithm, and calculated the final weights. The risk scores for debris flows were obtained by multiplying the final weights with the normalized debris flow data. The above process is shown in Fig 2.

## 4 Results

The weights of Tables 6–8 were brought into formulas (8), (9) (10) to calculate the weight intervals, and the results are shown in Table 10.

**Table 10:** Weight intervals at different confidence levels.

	50%	60%	70%	80%	90%	95%
X_1_	[0.195,0.204]	[0.194,0.205]	[0.193,0.206]	[0.191,0.208]	[0.188,0.211]	[0.185,0.214]
X_2_	[0.262,0.332]	[0.253,0.341]	[0.242,0.352]	[0.228,0.366]	[0.204,0.390]	[0.181,0.413]
X_3_	[0.051,0.100]	[0.045,0.106]	[0.037,0.114]	[0.027,0.124]	[0.011,0.140]	[-0.005,0.156]
X_4_	[0.103,0.112]	[0.102,0.114]	[0.101,0.115]	[0.099,0.117]	[0.096,0.120]	[0.093,0.123]
X_5_	[0.168,0.221]	[0.161,0.228]	[0.153,0.237]	[0.141,0.248]	[0.123,0.266]	[0.106,0.284]
X_6_	[0.096,0.155]	[0.088,0.163]	[0.078,0.173]	[0.066,0.185]	[0.046,0.205]	[0.026,0.225]

**Fig 3:**
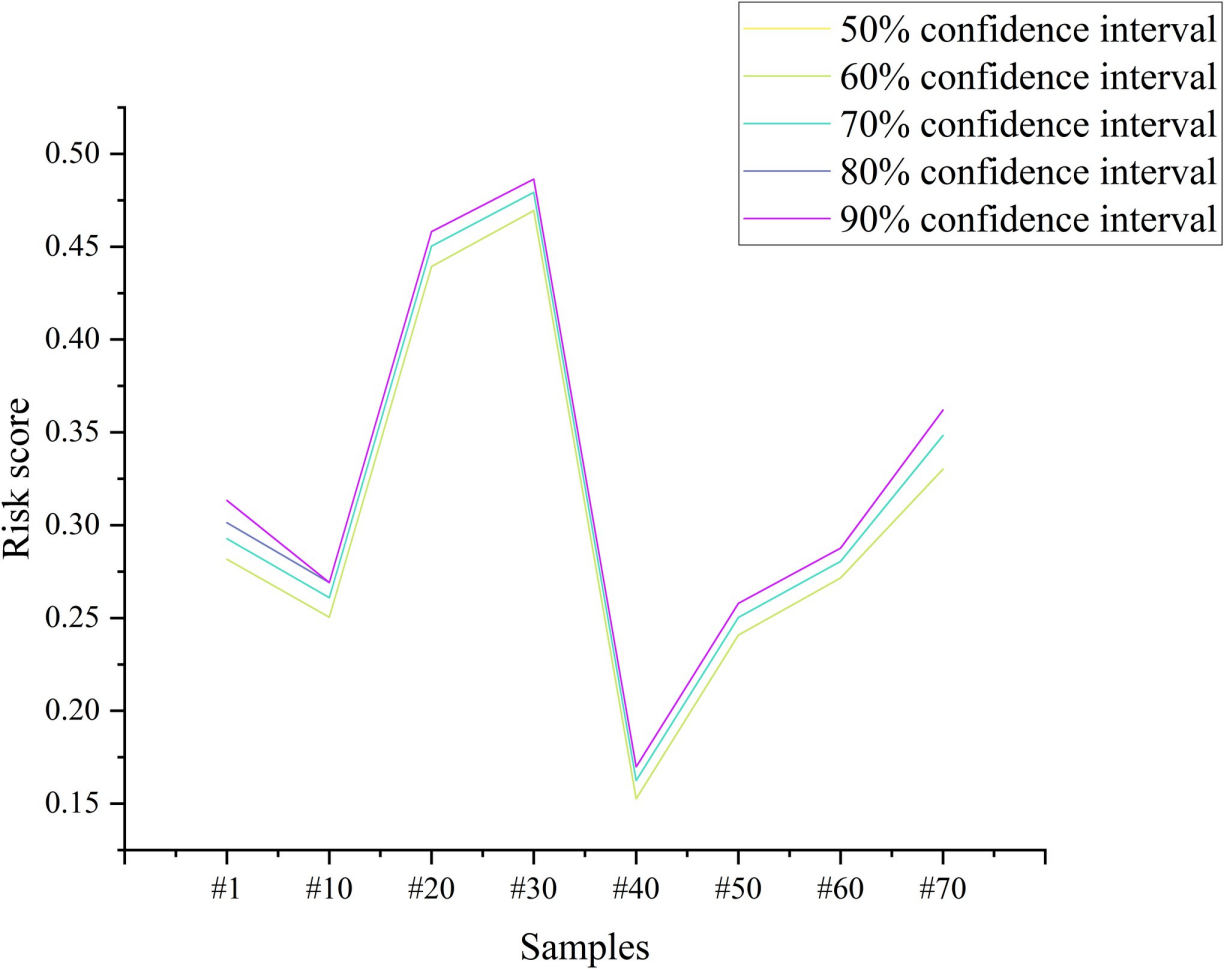
The risk scores of 8 samples at different intervals.

Before calculating the final weights using the LPOA, this paper selected the risk scores of 8 samples at different intervals to observe the results trend, as shown in Fig 3.

**Fig 4:**
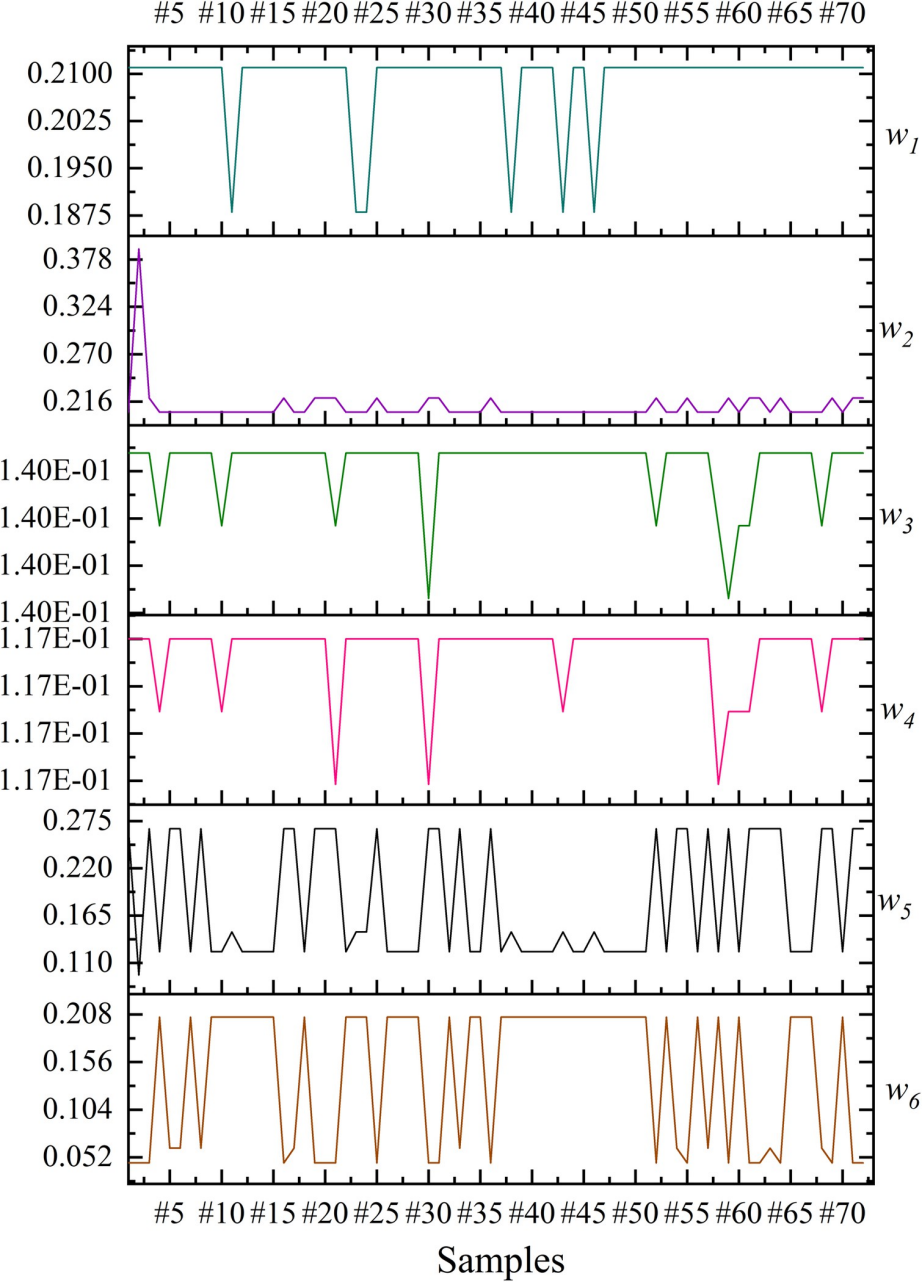
The risk scores of 8 samples at different intervals.

The results showed that the risk score for each debris flow increases as the confidence interval increased, but the trend is not significant. This paper did not calculate the weight values for the 95% confidence intervals. Because the weight of *X*_*3*_ may be <0 in that interval. However, the definition of weights needs satisfying that all weights are > 0 [14]. But the reliability of models increases with the confidence level. Therefore, this paper selected the 90% confidence interval to calculate the final weights, and the results are shown in Fig 4. Where *w*_1_ are the weights of *X*_*1*_, and the others are the same.

The results showed that the weights obtained under the constraints are dynamic. The weights of the assessment metrics for each debris flow gully are distinctive. For example, the basin area (*X*_*2*_) of the #2 debris flow was given a greater weight. Because the basin area of #2 debris flow is comparatively large compared to other debris flows of the same size. Similarly, the debris flow scale (*X*_*2*_) of #11 debris flow was given a smaller weight.

The final weights from Fig 4 were brought into formula (13) to calculate the risk scores. The risk scores of the proposed method, AHP, EWM, VCM and CWPA were sorted as shown in Table 11.

**Table 11:** Risk score sorting of proposed methods, AHP, EWM, VCM, and CWPA.

Sorting	Proposed method	CWPA	AHP	EWM	VCM
1	#19	#19	#19	#69	#69
2	#69	#69	#64	#19	#19
3	#64	#64	#69	#64	#64
4	#16	#16	#16	#16	#16
5	#55	#55	#25	#55	#55
6	#31	#31	#31	#52	#52
7	#25	#25	#55	#31	#31
8	#52	#52	#52	#25	#25
9	#59	#59	#2	#59	#59
10	#36	#36	#32	#36	#36
11	#2	#2	#59	#2	#2
12	#32	#72	#36	#72	#72
13	#72	#61	#33	#61	#61
14	#61	#30	#18	#30	#30
15	#21	#32	#61	#21	#21
16	#30	#21	#30	#20	#32
17	#33	#33	#21	#71	#20
18	#6	#6	#72	#6	#71
19	#18	#20	#6	#32	#33
20	#20	#18	#71	#33	#6
21	#71	#71	#20	#3	#18
22	#3	#3	#3	#18	#3
23	#8	#8	#14	#62	#62
24	#62	#62	#4	#8	#8
25	#14	#14	#8	#14	#14
26	#4	#4	#62	#4	#4
27	#17	#17	#17	#17	#17
28	#11	#68	#11	#68	#68
29	#70	#57	#70	#57	#57
30	#68	#63	#68	#63	#63
31	#57	#70	#57	#15	#15
32	#63	#11	#63	#22	#70
33	#15	#22	#7	#70	#22
34	#22	#15	#15	#1	#11
35	#7	#38	#22	#56	#53
36	#38	#1	#38	#54	#1
37	#9	#53	#9	#53	#51
38	#53	#7	#53	#60	#56
39	#51	#56	#51	#51	#38
40	#1	#9	#1	#38	#60
41	#37	#37	#37	#5	#54
42	#56	#67	#56	#11	#67
43	#67	#51	#67	#67	#65
44	#60	#54	#60	#65	#9
45	#65	#60	#65	#37	#37
46	#54	#65	#34	#9	#5
47	#58	#58	#58	#7	#7
48	#34	#5	#45	#10	#58
49	#45	#10	#41	#58	#10
50	#41	#41	#54	#44	#44
51	#35	#44	#35	#50	#50
52	#47	#66	#47	#49	#49
53	#10	#50	#10	#66	#41
54	#26	#49	#26	#41	#47
55	#5	#47	#66	#47	#66
56	#49	#34	#49	#34	#34
57	#66	#35	#50	#35	#35
58	#50	#45	#44	#27	#45
59	#44	#26	#5	#45	#27
60	#43	#27	#48	#26	#26
61	#48	#48	#43	#48	#48
62	#27	#43	#27	#29	#29
63	#29	#29	#29	#24	#43
64	#24	#24	#24	#43	#24
65	#39	#39	#39	#39	#39
66	#28	#46	#28	#23	#28
67	#46	#28	#46	#28	#23
68	#23	#23	#23	#46	#46
69	#13	#13	#13	#13	#13
70	#12	#40	#12	#40	#40
71	#40	#12	#40	#12	#12
72	#42	#42	#42	#42	#42

The results showed that each method had little difference in the results for sorting. The proposed methods, CWPA, and AHP considered #19 debris flow to be the most dangerous, while EWM and VCM considered #69 debris flow to be the most dangerous. However, the proposed method and CWPA puts #69 in 2nd place, while the AHP puts #69 in 3rd place. Because the EWM and VCM underestimated the effect of debris flow frequency (*X*_*6*_) on debris flow risk. The weight of *X*_*6*_ calculated by EWM is 0.066, and the weight of *X*_*6*_ calculated by VCM is 0.103. However, the analysis in section 2.2 showed that the debris flow frequency is one of the most directly influential indicators for assessing debris flow risk [14]. Therefore, due to calculating weights only based on data relationships, the objective decision-making methods may be unreliable. In contrast, the proposed method improves the reliability of objective decision-making methods by combining the weights of AHP, EWM and VCM. For #42 debris flow, each method puts it in 72nd place. The data showed that the #42 debris flow scale, basin area, basin cutting density, basin relative elevation difference, main gully length, and the occurrence frequency are 9.65×103m^3^, 0.3km^2^, 10.67km^-1^, 0.52km, 0.66km, and 10, respectively. Compared to other debris flows, the topography and hydrologic conditions of #42 debris flow cannot provide favorable conditions for high-risk debris flows. The CWPA and AHP put #40 in 71st place, while the proposed method, EWM and VCM put #40 in 70th place. The reasons for the variance are essentially similar to above analysis.

**Fig 5:**
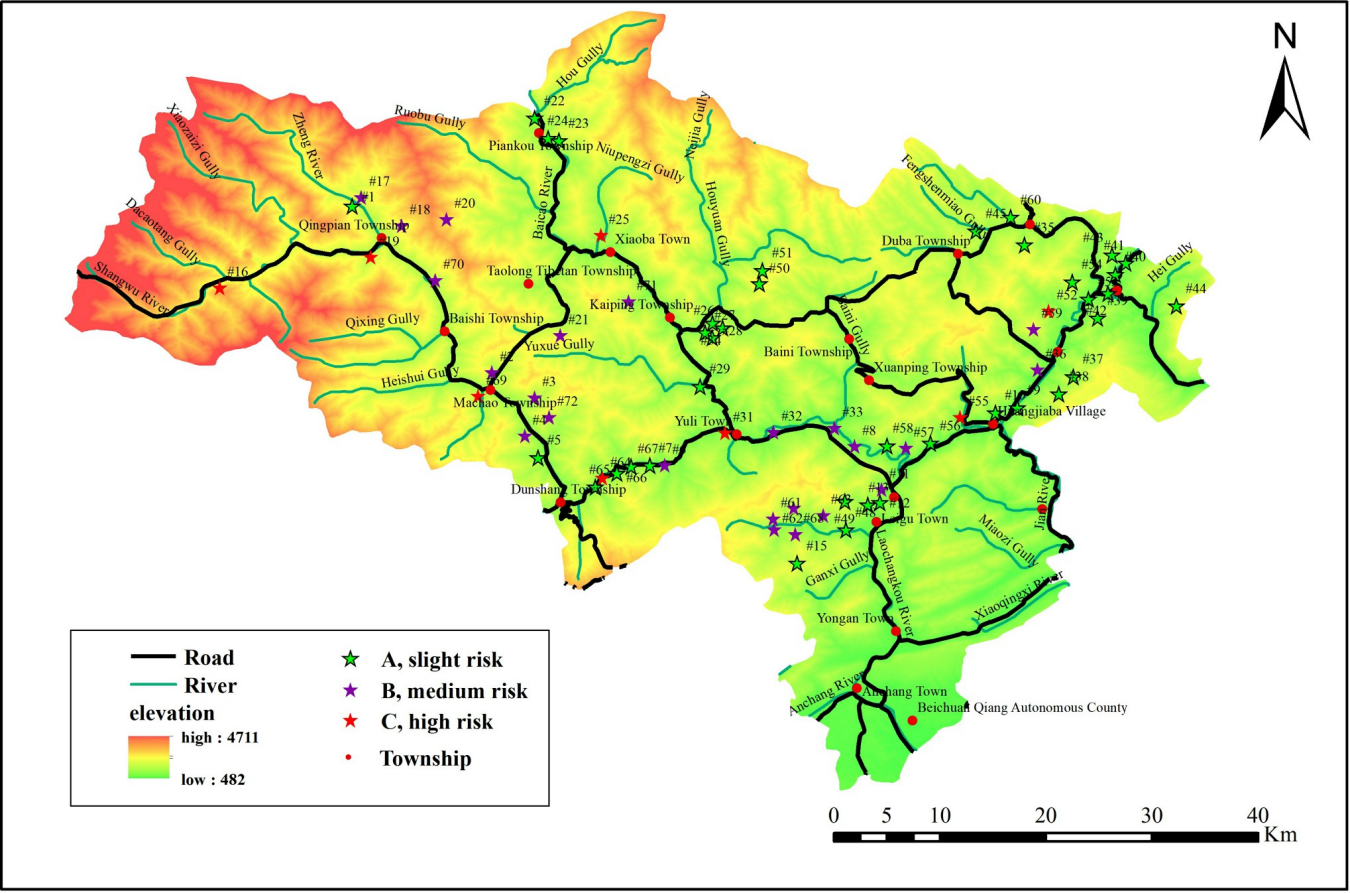
A combination weighting method for debris flow risk assessment based on t-distribution and linear programming optimization algorithm.

This paper determined the risk level of each debris flow based on Table 9, and the results are shown in Fig 5. The elevation data in this figure is the DEM at 12.5m.

The results showed that there are 8 high risk debris flows, including #19, #69, #64, #16, #55, #31, #25, and #52. Among them, #19, #69, #16 and #25 are distributed in the same area. The area is located at the junction of the Tibetan Plateau and the Sichuan Basin, with large elevation differences and unstable geological structures. Therefore, these 4 debris flows are assessed as high risk. The data showed that the volume of loose deposits in #64, #55, #31, and #52 are 378.24×10^3^m^3^, 114×10^3^m^3^, 900.03×10^3^m^3^, and 98.5×10^3^m^3^, respectively [14]. And all 4 debris flows are located near rivers, with good hydrological conditions and material reserves providing favorable conditions for larger debris flows. Therefore, these 4 debris flows are also assessed as high risk. In addition, there are 24 medium risk debris flows and 40 slight risk debris flows. These debris flows are mainly located on the sides of highways and rivers. Although they are not as dangerous as high-risk debris flows, they can still pose a considerable threat to Beichuan County.

## 5. Discussions

**Fig 6:**
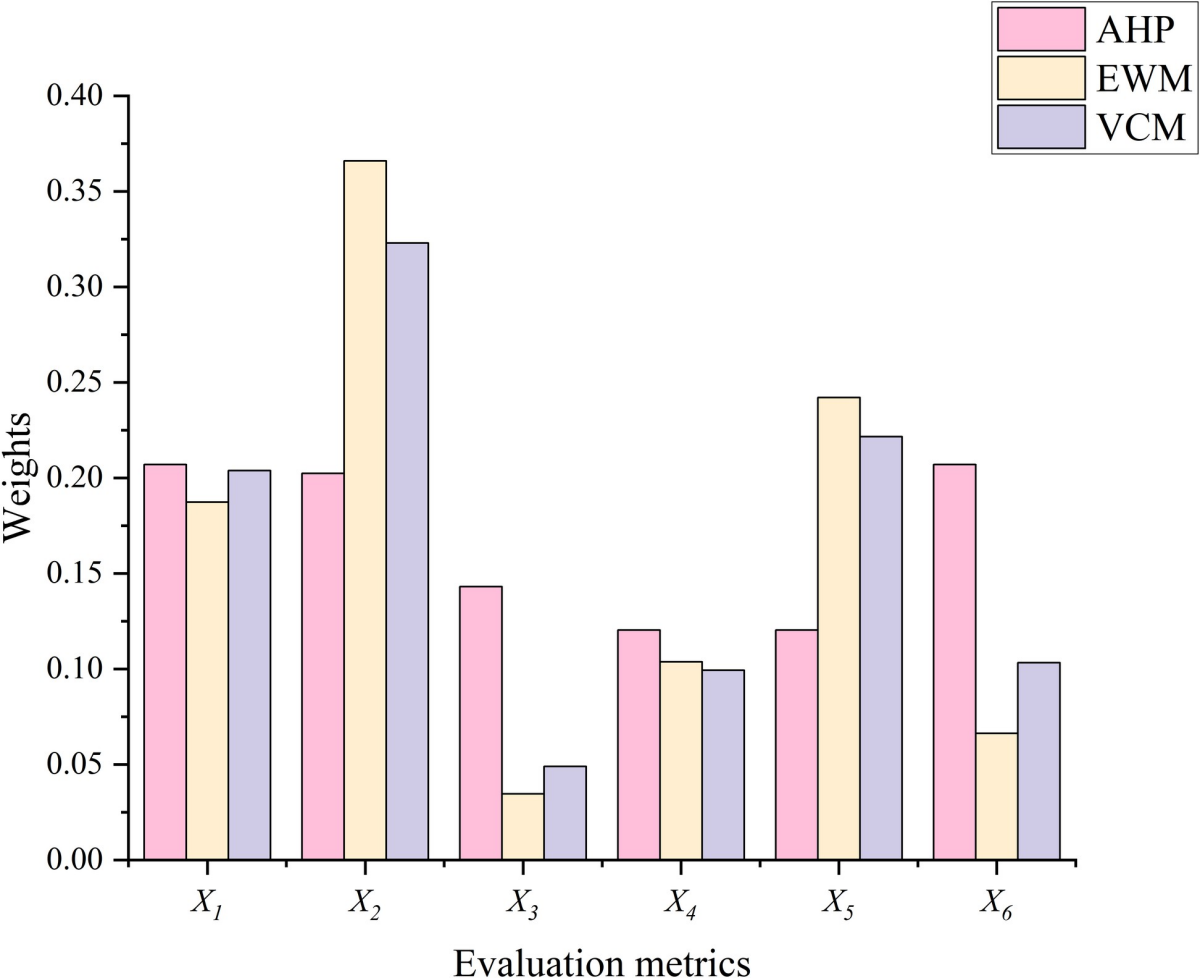
The weights of AHP, EWM and VCM.

The weights of each assessment metric calculated by AHP, EWM and VCM were counted, and the results are shown in Fig 6.

The results showed that subjective and objective decision-making methods differ in some assessment metrics. The objective decision-making method reflects the metric weights mainly through the degree of data variability. The basin area (*X*_*2*_) in the study area has a maximum value of 26.4 km^2^ and a minimum value of 0.3 km^2^. In contrast, the debris flow frequency (*X*_*6*_) has a maximum value of 96 times per 100 years and a minimum value of 10 times per 100 years. Therefore, both EWM and VCM considered *X*_*2*_ as the main factor influencing the debris flow risk. However, both research [14] and field investigation results showed that the *X*_*6*_ is one of the indicators that most directly affects the debris flow hazard. Consequently, EWM and VCM ignored the importance of this metric. Due to the advantages and disadvantages of both methods, this paper fused the weights of AHP, EWM and VCM through t-distribution. Fusion can avoid overemphasizing the importance of a specific metric. In addition, the fused weights can consider both the field survey’s perceptions and the laws of objective data. The weight obtained by CWPA in an interval is an average value. In contrast, LOPA can obtain dynamic weights based on the characteristics of the debris flow. For example, the basin area (*X*_*2*_) of #2 debris flow is larger compared to other debris flows of the same size, thus the LOPA assigns a larger weight to it. LOPA can provide a more refined assessment for each debris flow than the average weights of the CWPA. In addition, the t-distribution can produce different intervals at different confidence levels (Table 10). The results showed that the final weights in this paper increase as the confidence interval increasing (Fig 3). This means that the actual operator is allowed to choose different confidence intervals depending on the actual situation.

**Fig 7:**
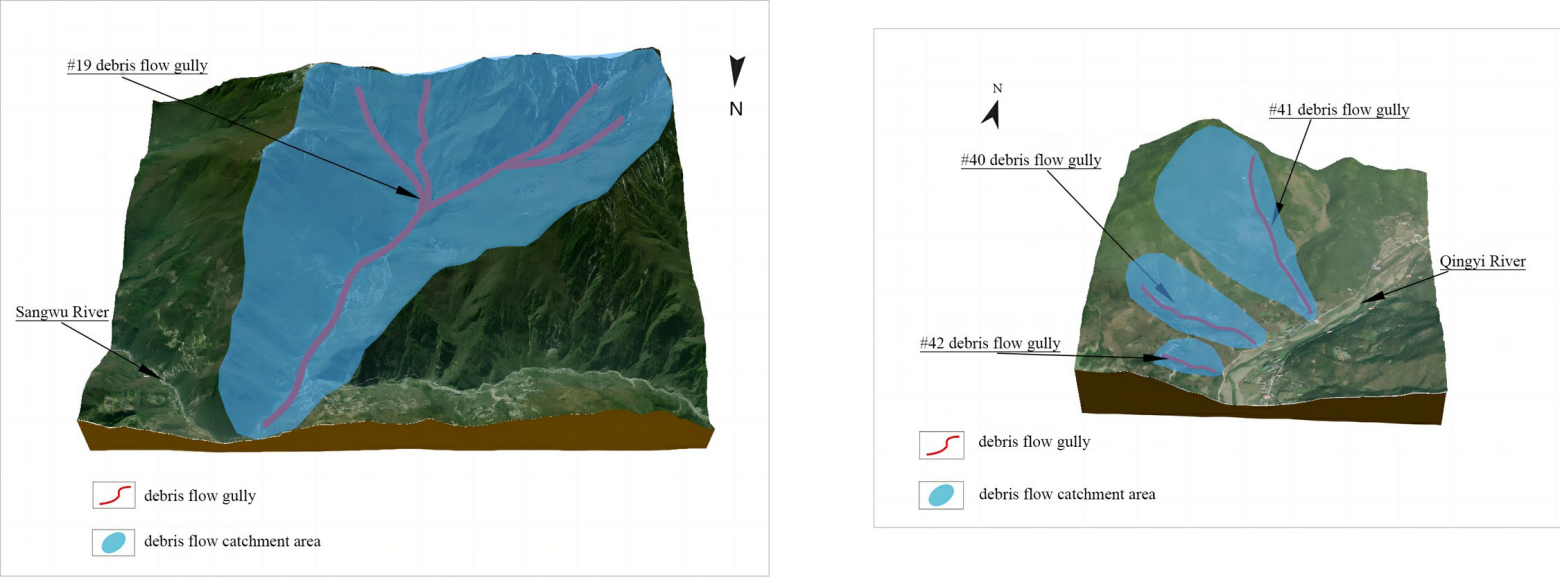
#19, #40, #41, and #42 Debris flow 3-D topography. (a) #19 debris flow (b) #40, #41, and #42 debris flows.

Due to the large amount of sample data, this paper selected #19 debris flow with the highest risk score and #40, #41, and #42 with lower risk scores for analysis based on the sorting results in Table 11. The Fig 7showed the 3-D topographic maps of each debris flow.

The comparison in Fig 7 showed that key factors of #19 debris flow such as scale, catchment area, and main ditch length consisting of multiple tributaries are much larger than the other debris flows. Its actual scale, catchment area, and main ditch total length are 152.47×10^3^m^3^, 23.2km^2^, and 7.49km, respectively. And #19 debris flow is in the western part of Beichuan County and the junction of the Tibetan Plateau and the Sichuan Basin. Frequent geologic activity causes increased instability, so #19 calculations to get the highest risk score are consistent with reality. From the sorting results, the sorting sequence of #40, #41, and #42 are 71, 50, and 72, respectively. The scale of #41 debris flow is 28.98×10^3^m^3^ (about twice as large as #40) with a catchment area of 2.2 km^2^ (about three times as large as #40). Therefore, the reasonableness of the sorting results is verified. In addition, the scale and catchment area and main gully length of #40 and #42 debris flows are much smaller than other debris flows in the area. Moreover, the 2 debris flow gullies are in the Sichuan Basin east of the study area, with stable geologic conditions and low relative elevation differences. The reasonableness of the proposed method is verified by comparison analysis and actual data.

Although this paper verifies the reasonableness of the proposed method for 72 debris flows in Beichuan County after comparative analysis and actual data, there are still some limitations in this paper:

1. The weights calculated by AHP are better reflecting the intuitive perception of the field survey compared to the equal weighting method. However, the AHP is a subjective decision-making method based on expert scoring. This paper uses objective decision-making methods to undermine this subjectivity to a certain extent, but it is still important to pay attention to the reasonableness of the AHP results in the process of using it.
2. In order to compare the results for reasonableness, this paper only uses 6 extant assessment metrics. However, the study area of this paper had been affected by aftershocks several times since the Wenchuan mega-earthquake. There were 5 earthquakes of different magnitudes on October 21–23, 2020, alone. Therefore, using the earthquake intensity index as an assessment factor in future studies may achieve a more accurate assessment of debris flow hazard in the area.
3. The final risk score increases as the confidence interval increasing. This paper selected intervals that balance accuracy and reliability as well as satisfy the definition of weights. But how to select the appropriate interval according to the actual engineering situation is still a problem that needs to be explored. In addition, the uncertainty in the debris flow risk analysis still needs to be further investigated. For example, uncertainty measures such as approximate entropy and correlation coefficients can be used to quantify and manage uncertainty in risk analysis [29, 30].
4. Combining remote sensing data to determine the risk level of roads and villages threatened by debris flow can provide a reliable reference for debris flow risk assessment. For example, Tang et al. [31] conducted the dynamic analysis of debris flows based on remote sensing of Beichuan County. Therefore, future work may consider integrating remote sensing data to achieve more reliable debris flow risk assessment.

## 6. Conclusions

This paper proposed a method for determining the weights based on t-distribution and linear programming optimization algorithm. The proposed method was applied to 72 debris flows in Beichuan County for risk assessment. After comparison analysis and actual data for validation, the following main conclusions were obtained:

1. The debris flow frequency is one of the assessment indicators that most directly affects the risk of debris flows. However, the entropy weight method and variation coefficient method ignored this metric’s effect on debris flow risk. The proposed method by fusing weights simultaneously considered the researcher’s intuitive perception in the field survey and respected the laws of objective data. The linear programming algorithm can take dynamic values based on the data characteristics of debris flows. The sorting of risk scores calculated by this method did not differ significantly from other methods, thus validating the rationality of the method. The risk scores at different confidence levels increase as the confidence level increasing.
2. The 72 debris flows in Beichuan County are dominated by medium and light risks. Among them, there are 8 high-risk debris flows. #19, #69, #16, and #25 are characterized by intensive geologic activity, large topographic elevation differences, and extensive watersheds. And #64, #55, #31, #52 have a lot of loose material and close to the river. In addition, there are 24 mid-risk debris flows and 40 light-risk debris flows. These debris flows are mainly located on both sides of highways and rivers, and still cause a considerable threat to Beichuan County.
3. In future studies, considering the rationalization of AHP results and more assessment metrics can obtain more accurate assessment results. In addition, how to select appropriate confidence intervals according to actual engineering conditions and eliminating the uncertainty effects are issues that still need to be explored.

## Supporting information

S1 File (RAR)
